# X-Ray Crystallographic Analysis, EPR Studies, and Computational Calculations of a Cu(II) Tetramic Acid Complex

**DOI:** 10.1155/2017/7895023

**Published:** 2017-02-19

**Authors:** Dimitrios Matiadis, Dimitrios Tsironis, Valentina Stefanou, Olga Igglessi–Markopoulou, Vickie McKee, Yiannis Sanakis, Katerina N. Lazarou, Athanassios Chrissanthopoulos, Spyros N. Yannopoulos, John M. Markopoulos

**Affiliations:** ^1^National Technical University of Athens, School of Chemical Engineering, Laboratory of Organic Chemistry, 15773 Athens, Greece; ^2^School of Chemical Sciences, Dublin City University, Glasnevin, Dublin 9, Ireland; ^3^Institute of Nanoscience and Nanotechnology, NCSR Demokritos, Ag. Paraskevi, 15310 Attiki, Greece; ^4^Laboratory of Inorganic Chemistry, Department of Chemistry, National and Kapodistrian University of Athens, Panepistimiopolis, 15771 Athens, Greece; ^5^Foundation for Research and Technology Hellas, Institute of Chemical Engineering Sciences (FORTH/ICE-HT), P.O. Box 1414, Rio, 26504 Patras, Greece

## Abstract

In this work we present a structural and spectroscopic analysis of a copper(II) N-acetyl-5-arylidene tetramic acid by using both experimental and computational techniques. The crystal structure of the Cu(II) complex was determined by single crystal X-ray diffraction and shows that the copper ion lies on a centre of symmetry, with each ligand ion coordinated to two copper ions, forming a 2D sheet. Moreover, the EPR spectroscopic properties of the Cu(II) tetramic acid complex were also explored and discussed. Finally, a computational approach was performed in order to obtain a detailed and precise insight of product structures and properties. It is hoped that this study can enrich the field of functional supramolecular systems, giving place to the formation of coordination-driven self-assembly architectures.

## 1. Introduction

**Scheme 1:**
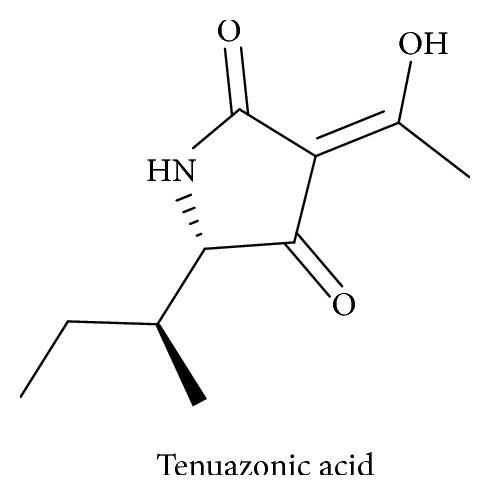


**Scheme 2:**
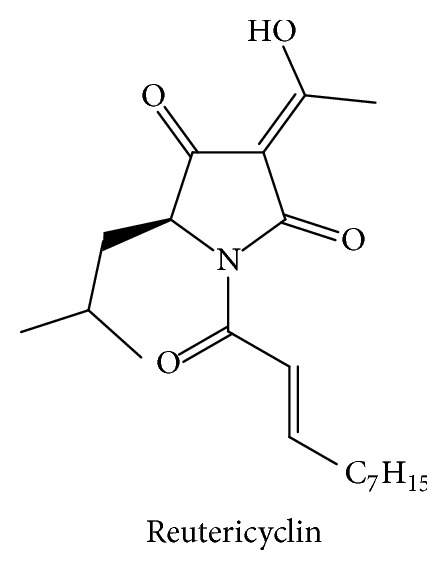


**Scheme 3:**
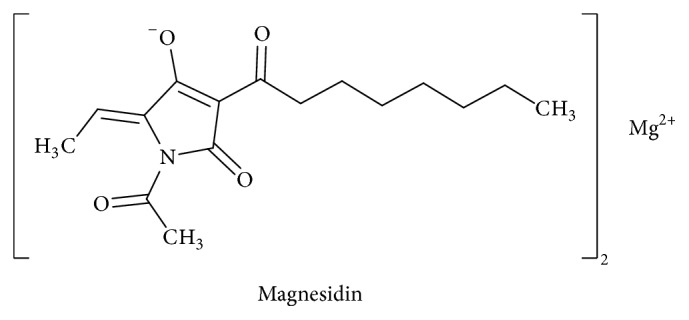


**Scheme 4:**
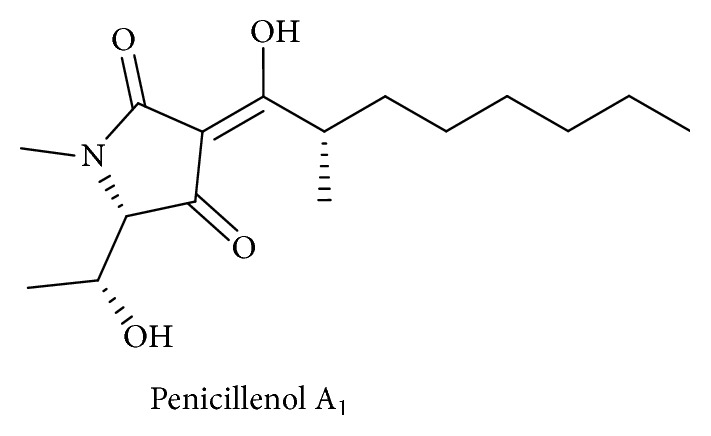


Tetramic acid derivatives (pyrrolidine-2,4-diones) constitute a unique class of nitrogen five-membered heterocyclic compounds that have attracted significant attention over the years due to their occurrence in naturally bioactive materials. They present various pharmaceutical and biological activities including antibiotic, cytotoxic, antifungal, and anti-HIV activities [1–5]. Representative biologically active natural products containing the 2,4-pyrrolidinedione system include tenuazonic acid (Scheme 1) [6, 7], reutericyclin (Scheme 2) with a wide range of pharmacological activities [8–10], the HIV integrace inhibitor equisetin and the tetramic acid-homologues possessing the unsaturated decalin ring system [11], the antibiotic “Magnesidine” (Scheme 3) containing the 5-ethylidene and 3-alkanoyl substitutes with Mg(II) [12, 13], harzianic acid, showing Iron (III)-binding affinity [14, 15], penicillenols (Scheme 4) [16, 17], epicoccamides [18], streptolydigin which inhibits RNA polymerase [19, 20], and the melophlin family of compounds which have shown antimicrobial activity [21].

The 3-acyl substituted tetramic rings provide metal binding capacity [22, 23], and this system has been primarily investigated in the fungal metabolite tenuazonic acid which has shown to form complexes with Ca(II) and Mg(II) [24]. Recently, the naturally occurring “chaunolidines A–C” tetramic acid analogues, isolated from an Australian marine-derived fungus, were shown to form metal chelates with Fe(III), Au(II), Cu(II), Mg(II), and Zn(II) [25]. Melophlin, a member of N-methyl-3-acetyl tetramic acid, chelates with Mg, Zn, Ga, La, and Ru ions [21, 26]. Biological evaluation of the complexes shows antiproliferative activity against various cancer cells. Additionally, more complexes of naturally derived tetramic acids have been reported, the most important being those of decylidene tetramic acid (C12-TA), a degradation product derived from 3-oxododecanoyl homoserine lactone (3-oxo-C_12_-HSL) that binds essential metals such as Fe(III) and Ga(III) ions [27]. Recently, a new synthetic methodology on tetramic acid core and subsequent organocesium carbanionic architectures has been explored [28]. Likewise, the synthesis of chelating agents containing the oxygen equivalent 3-acyltetronic acid scaffolds has been extensively studied [29].

Construction of densely functionalized 3-acyltetramic acids is a topic of continuing interest. The development of effective synthetic methodologies and theoretical investigations on the tautomerism of these compounds have been the focus of many researchers [30–32].

Our research group has contributed notably to the synthesis and study of five-membered *β*,*β*′-tricarbonyl oxygen and nitrogen heterocycles and their coordination compounds. The *β*,*β*′-tricarbonyl compounds form a diverse class of ligands with many applications in inorganic chemistry. The 3-acylated tetramic acids (heterocyclic core), in their deprotonated form, act as ligands for the synthesis of coordination compounds. The 5-arylidene-3-alkanoyl tetramic acids contain structural adjuncts, an enolic *β*,*β*′-tricarbonyl moiety, a lipophilic 3-alkanoyl moiety, and a hydrophobic group at the 5-position anticipated to permit versatile activity. The ability of N-acetyl-tetramic acids as chelating monoanions prompted us to investigate the synthesis of rhodium(I) [33], cationic diammineplatinum(II) complexes [34], and palladium (II) complexes [35]. Additionally, we have investigated the coordination ability of tetramic acids with transition metal ions such as copper(II) nickel(II), cobalt(II), and zinc(II) [36]. Here, our interest has been focused on the further study on the recently presented copper(II) and zinc(II) complexes of N-acetyl-5-arylidene tetramic acid [37].

Copper(II) ion has been found in many supramolecular features [38] and metalloproteins [39]. Copper and its compounds have many medical applications. Copper(II) complexes have been used as analgetic, antipyretic, anti-inflammatory, and platelet antiaggregating agents. They have antioxidant activity and protect against the consequences of UV exposure. Binuclear complexes like Cu_2_(asp)_4_  {asp = aspirinate} exert additional activities, including antiulcer, anticancer, antimutagenic, and antimicrobial effects [40]. Copper(II) complexes may have less severe side effects and may overcome acquired and inherited resistance to medicines based on platinum(II) [41, 42].

Recently, it has been reported that copper (II) complexes incorporating ligands with appended functional groups can be used in early AD (Alzheimer Disease) pathology by PET (position emission tomography). Clinical studies for Cu(II) complexes with a functionalized styrylpyridine group indicate binding to amyloid-*β* plaques and effectively crossing of the blood-brain barriers [43]. Consequently, transition metals, such as Cu and Zn, have a suggested link to AD pathology.

In this report we present the crystal structure and EPR spectroscopic properties of the copper(II) complex. Computational calculations on the complex were also performed in order to obtain a detailed and precise insight into the structure and properties of the complex.

## 2. Experimental Section

### 2.1. Materials and Methods

The ligand, 3-acetyl-5-benzylidene-tetramic acid, and the copper (II) complex were prepared according to our previous publication [37].

### 2.2. Synthesis of [Cu(TA-H^+^)_2_(EtOH)_2_]

To a solution of 3-acetyl -5-benzylidene tetramic acid (TA) (1.1 mmoL) in the minimum amount of ethanol was added Cu(CH_3_COO)_2_·H_2_O (0.55 mmoL), dissolved in the minimum amount of ethanol, and the resulting solution was refluxed under stirring for 2 hours. The reaction mixture was left to cool at rt, and the precipitate was filtered, washed with ethanol, and dried to give a pale green solid (374 mg, 98%), mp 173°C (dec), *λ*_max_ (CHCl_3_)/nm 341 (log⁡*ε* 4.46) and 642 (2.51), *ν*_max_/cm^−1^ 3520 (s), 1740 (s), 1690, 1590 (s), 1490 (s), 1370, 490 (w), HRMS: calcd for C_30_H_25_N_2_O_8_Cu 604.0908; found 604.0807.

### 2.3. X-Ray Crystallography

Crystals of [Cu(TA-H^+^)_2_]·2EtOH suitable for X-ray crystallography were obtained from a solution of ethanol diffused with diethyl ether.

The data were collected at 150(2) K on a Bruker-Nonius Apex II CCD diffractometer using Mo*K*_*α*_ radiation (*λ* = 0.71073 Å) and were corrected for Lorentz-polarization effects and absorption. The structure was solved by direct methods and refined on *F*^2^ using all the reflections [44] All the nonhydrogen atoms were refined using anisotropic atomic displacement parameters and hydrogen atoms were inserted at calculated positions using a riding model. Crystal data, data collection, and structure refinement details are summarised in Table 1. “CCDC nnnnnnn contains the supplementary crystallographic data for this paper. These data can be obtained free of charge from The Cambridge Crystallographic Data Centre via https://www.ccdc.cam.ac.uk/structures-beta/.”

### 2.4. EPR Spectroscopy

CW EPR measurements at Q-band were carried out on a home-assembled spectrometer equipped with an ER 5106 QT Bruker resonator. Simulations of the spectra were performed with the SpinCount software kindly provided to us by Professor Michael Hendrich, Carnegie Mellon University, Pittsburgh, PA, USA.

### 2.5. Computational Methods and Details

Density functional theoretical methodology was selected for the calculation of the structural details of the tetramic acid (ligand) and metal complexes' structural models shown in Figures 4 and 5 and Table 3. The B3LYP [45–47], the most widely used of all the functionals, was employed as implemented in the GAUSSIAN 09 program package [48].

The accuracy and good performance of the computational methods and basis sets, which are chosen for the investigated systems, have been tested by comparing the calculated properties with experimental structural data that are available in the literature [37].

The electronic structures of the atoms participating in the investigated structures are described by the Ahlrichs triple zeta TZVP basis sets included in definition polarization Gaussian-type functions (GTF) [49, 50]. The geometries of the structural models of the ligand and the complexes have been fully optimized at the B3LYP/TZVP level of theory using the Berny algorithm as implemented in Gaussian 09 program package. All optimized geometries correspond to stationary points on the potential energy surface, as no imaginary frequencies have been obtained.

The calculation of natural atomic charges was performed with the natural bond orbital, NBO 3.1 program [51] on optimized geometries at B3LYP level of theory with the TZVP basis set.

## 3. Results and Discussion

### 3.1. Structure of [Cu(TA-H^+^)_2_·2EtOH]

**Figure 1:**
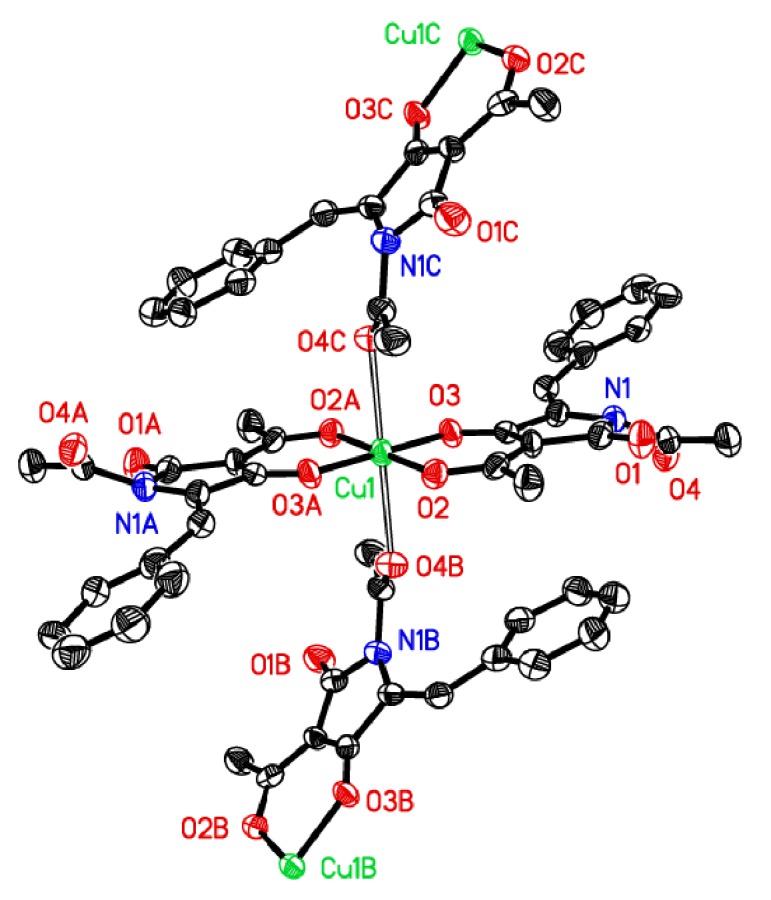
Perspective view of the complex showing 50% probability ellipsoids. Suffixes on the atom labels indicate atoms generated using the following symmetry operations: A, −*x*, 2 − *y*, −*z*; B, −*x*, 1/2 + *y*, 1/2 − *z*; C, *x*, 3/2 − *y*, −1/2 + *z*. Hydrogen atoms and the ethanol solvate molecule omitted for clarity.

**Figure 2:**
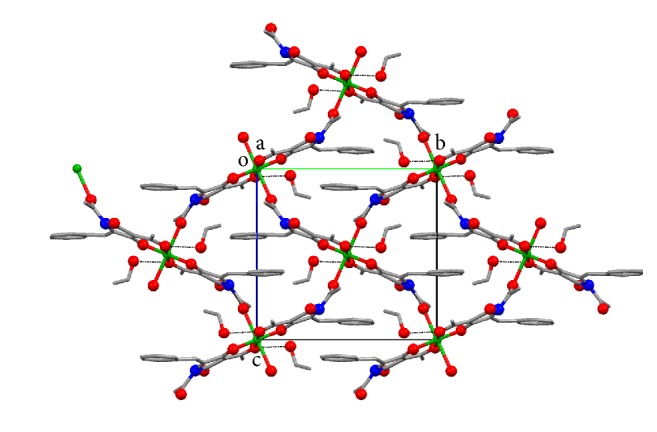
Part of the polymeric sheet structure parallel to the *a*-axis. Hydrogen bonds shown as dashed lines; hydrogen atoms omitted for clarity.

The copper ion lies on a centre of symmetry (Figure 1), so the asymmetric unit comprises one ligand molecule, one ethanol solvate molecule, and half of a copper(II) ion. The geometry at the metal ion is tetragonal; the copper ion is coordinated to two bidentate (TA-H^+^) anions in the basal plane and,* via* longer apical bonds (2.522 (4) Å), to the amide oxygen atoms of two neighbouring complexes. Each ligand ion is therefore coordinated to two copper ions, forming a 2D sheet lying perpendicular to the *a*-axis (Figure 2). There are no notable interactions between the sheets.

The ethanol solvate molecules are hydrogen bonded to one of the oxygen donors in the square plane (O5–H5⋯O2; 3.010 (6) Å under symmetry operation* x*, −*y* + 3/2, *z* + 1/2) and have a longer interaction with the other (O5–H5⋯O3; 3.213 (6) Å under −*x*, *y* − 1/2, –*z* + 1/2). The mean plane of the ligand amide group makes an angle of 35.3 (2)° with the mean plane of the five-membered ring. Selected bond lengths and angles are given in Table 2.

### 3.2. EPR Spectroscopy

**Figure 3:**
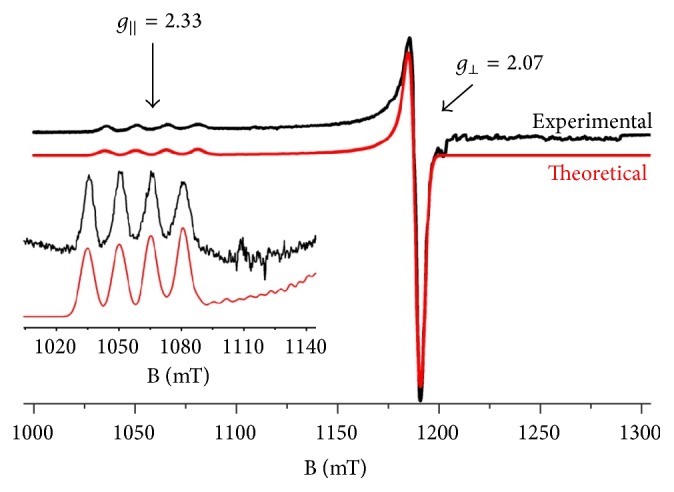
Experimental (black line) and theoretical (red line) Q-band EPR spectra from a frozen chloroform solution of Cu(II) complex. The inset focuses on the *g*_||_ part of the spectrum. EPR conditions: temperature, 100 K; modulation amplitude, 5 Gpp; microwave power, 1.3 mW; microwave frequency, 34.4 GHz.

The EPR (Figure 3) shows the Q-band EPR spectrum from a frozen solution of Cu(II) complex in chloroform recorded at 100 K. The spectrum consists of a typical axial Cu^2+^ signal with *g*_||_ > *g*_⊥_. The *g*_||_ component splits further into four lines due to hyperfine interactions with the ^63/65^Cu (I = 3/2) nucleus. Simulation of the spectrum yields *g*_||_ = 2.33, *g*_⊥_ = 2.07, and *A*_||_ = 490 MHz. The values of *g*_||_ and *A*_||_ are in line with the unpaired electron being in *d*_*x*_^2^ _−*y*_^ ^^2^^ ^ orbital and the equatorial coordination mode of “**4** Oxygens” [52] in agreement with the crystal structure.

### 3.3. Computational Methods

#### 3.3.1. Calculated Structural Details

**Figure 4:**
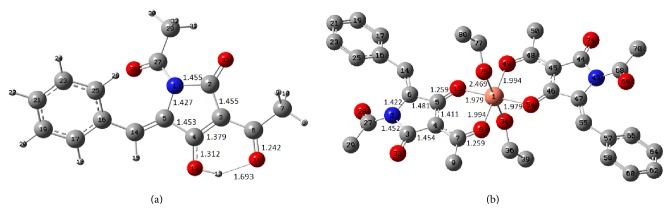
Ball and stick draws of the optimized structures: (a) N-acetyl-3-acetyl-5-arylidenetetramic acid (TA) and (b) copper(II) complex [Cu(TA-H^+^)_2_(EtOH)_2_]. In Figure (b) hydrogen atoms have been omitted for clarification. Main bond lengths and atoms' number are labeled. Calculations have been performed at B3LYP/TZVP level of theory.

The gas phase ligand molecule of tetramic acid (Figure 4(a)) is not planar as the calculated dihedral angle between the phenyl and pyrrole ring is about 31°, in accordance with the crystalline structure where there is a twist of 27.42 (8)° between the mean planes of the two rings. The centrosymmetric Cu(II)-complex (point group: C_i_), as presented in Figure 4(b), consists of two tetramic acid anions which act as bidentate ligands located in equatorial positions around the central metal. The two ligands are coordinated to the central metal via carbonyl oxygen bridges. The two –C=O– bonds are almost identical characterized by bond length of 1.259 Å (Table 3). The calculated dihedral angle between phenyl and pyrrole rings is about 25° which is in excellent agreement with the corresponding experimental value of the crystalline structure 25.13°.

Four oxygen atoms are positioned near the copper atom disposed in an almost square planar geometry. The two groups of diagonal oxygens have slightly different –O–Cu– bond distances, assuming values of 1.979 and 1.994 Å. In order to describe more realistically the solid state structure, two ethanol molecules were placed at axial positions. Therefore, the metal coordination sphere is completed by these two oxygen atoms from ethanol molecules in transpositions having a longer –O–Cu– distance, that is, 2.469 Å. The 2-3% variation between the calculated and crystallographic Cu-donor distances could be attributed to (i) the approximations adopted for the current structural model as two ethanol molecules have been placed in axial positions of the structural model instead of two other complexes mutually (up and down) arranged around the central complex in crystal's unit, (ii) crystal packing effects, and (iii) the theoretical level and basis set limitations of the calculations.

As expected there is an elongation of –C=O bond length upon complexation to the metal ion from 1.225 and 1.228 Å calculated for the ligand anion to 1.259 and 1.260 Å, for the copper and zinc complexes, respectively. These bonds have been measured at 1.268 and 1.253 Å [37] for copper and zinc complexes being very close to our calculations.

**Scheme 5:**
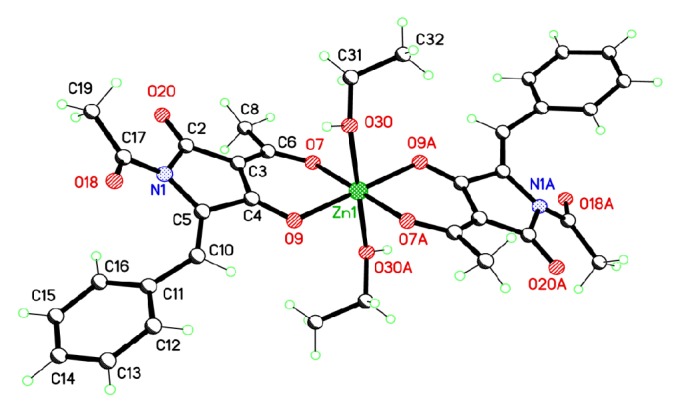


For comparison, in Table 3 equivalent crystallographic data for tetramic acid (TA) ligand and Zn(TA-H^+^)_2_·2EtOH (Scheme 5) are displayed [37], as well as selected calculated structural data at the B3LYP/TZVP level of theory.

#### 3.3.2. NBO Analysis

**Figure 5:**
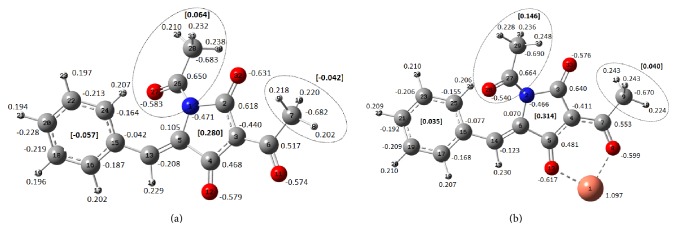
Atomic charges calculated by natural population analysis at B3LYP/TZVP level of theory of (a) N-acetyl-3-acetyl-5-arylidenetetramic anion (TA-H^+^) and (b) copper(II) complex (only a part of the structure is presented here). “Mean” charges of various groups are denoted as bold numbers in brackets.

Special attention was paid to donor-acceptor effects between ligand and metal. For this, natural bond orbital (NBO) theory and simulation have been applied [53–55]. The calculated natural atomic charges are presented in Figures 5(a) and 5(b) for the tetramic acid anion and the Cu-complex. Based on the calculated atomic charges we can estimate the “mean” charge of various groups (Figures 5(a) and 5(b)). Comparing the “mean” charge of various groups between the free anion and the complex it is revealed that a net electron density shift takes place towards the location of the Cu ion, which undergoes the greatest charge variation from +2 to +1.097.

Finally the binding energy for the Cu-complex has been calculated from the total energies of the optimized structures of the specific isolated species as

(1) EbindEcomplex−ECu2+−2Eligand  anion−2Eethanol=−3820.493227–−1639.4814993−2x−934.8883932−2x−155.104142=−1.0266573 Eh.

#### 3.3.3. Bond/Atom Valence Calculation

In bond-valence theory *S*_*ij*_, the valence of the *A*_*j*_ − *X*_*j*_ bond with the length *R*_*ij*_ is calculated via the following basic equation: *S*_*ij*_ = *e*^[(*r*_0_−*R*_*ij*_)/*b*]^, where *r*_0_ and *b* are empirically determined bond-valence parameters for a given cation-anion pair *A*, *X* [56]. According to this theory the atom valence *S*_Cu_  =  Σ(*S*_*ij*_).

In this study we used for [Cu–O pair] the parameters *r*_0_ = 1.679 and *b* = 0.36 [57].

The results are tabulated in Table 4.

## 4. Conclusions

In the present work we report a detailed investigation of a new Cu(II) complex involving the 3-acetyl-tetramic acid as “model ligand.” The structure and the “supramolecular” arrangement of the isolated complex have been investigated by single crystal X-ray crystallography. In addition, we have more completely characterized the complex with a combination of EPR studies and computational calculations. The consistency between theoretical and experimental values is good in general.

The 3-acetyl-tetramate ligand, functionalized with the 5-benzylidene group, has been used to prepare with Cu(OCOCH_3_)_2_·2H_2_O in ethanol, the complex copper (II) N-acetyl-5-benzylidene tetramic acid [Cu(TA-H^+^)_2_·2EtOH]. Each ligand ion is coordinated to two copper ions, forming a polymeric 2D sheet lying perpendicular to the *α*-axis, without notable interactions between the sheets. This model is a promising system for the development of “metallosupramolecular” architectures.

Work in progress includes the design of novel synthetic crystalline “organic linkers” to construct coordination-driven self-assembly architectures.

## Figures and Tables

**Table 1:** Crystal data.

Chemical formula	C_30_H_24_CuN_2_O_8_·2(C_2_H_6_O)
*M*_*r*_	696.19
Crystal system, space group	Monoclinic, *P*2_1_/*c*
Temperature (K)	150
*a*, *b*, *c* (Å)	9.745 (4), 12.979 (5), 12.375 (5)
*β* (°)	94.206 (7)
*V* (Å^3^)	1561.0 (11)
*Z*	2
Radiation type	Mo *Kα*
*μ* (mm^−1^)	0.76
Crystal size (mm)	0.41 × 0.15 × 0.04
Absorption correction	Multi-scan *SADABS* v20012/1, Sheldrick, G. M., (2012)
*T*_min_, *T*_max_	0.613, 0.745
Number of measured, independent, and observed [*I* > 2*σ*(*I*)] reflections	12319, 2757, 1590
*R*_int_	0.129
*R*[*F*^2^ > 2*σ*(*F*^2^)], *wR*(*F*^2^), *S*	0.062, 0.169, 1.01
Number of reflections, number of parameters	2757, 218
Δ〉_max_, Δ〉_min_ (*e* Å^−3^)	0.36, −0.39

**Table 2:** Selected bond lengths (Å) and angles (°).

Cu1–O3	1.930 (3)	Cu1–O4^ii^	2.522 (4)
Cu1–O2	1.936 (3)		

O3^i^–Cu1–O3	180.00 (18)	O2–Cu1–O2^i^	180.0
O3^i^–Cu1–O2	85.88 (13)	O3^i^–Cu1–O4^ii^	91.32 (13)
O3–Cu1–O2	94.12 (13)	O3–Cu1–O4^ii^	88.68 (13)
O3^i^–Cu1– O2^i^	94.12 (13)	O2–Cu1–O4^ii^	87.78 (13)
O3–Cu1– O2^i^	85.88 (13)	O2^i^–Cu1–O4^ii^	92.22 (13)

Symmetry codes: (i) −*x*, −*y* + 2, −*z*; (ii) −*x*, *y* + 1/2, −*z* + 1/2.

**Table 3:** Bond lengths in Angstroms of tetramic acid (TA) and Cu, Zn-complexes (Cu(TA-H^+^)_2_(EtOH)_2_), (Zn(TA-H^+^)_2_(EtOH)_2_) calculated by B3LYP method using the TZVP basis set. Atoms numbering refers to Figure 4.

	Complexes-bond length [Å]					Bonds	Ligand-bond length [Å]	
	Bonds	Cu-complex/B3LYP	Cu-complex/X-rays	Zn-complex/B3LYP	Zn-complex/X-rays [37]		B3LYP	X-rays [37]
						O12–H13	1.0043	1.0809

–N–C– bonds	N2–C6	1.4223	1.428	1.4208	1.4261	N1–C5	1.4269	1.4326
	N2–C27	1.4191	1.420	1.4183	1.3951	N1–C27	1.4224	1.4190
	N2–C3	1.4518	1.449	1.4526	1.4517	N1–C2	1.4549	1.4353

–C=O bonds	C7–O8	1.2588	1.281	1.2566	1.2534	C6–O11	1.2423	1.2592
	C27–O28	1.2102	1.214	1.2105	1.2219	C27–O28	1.2088	1.2124
	C3–O33	1.2134	1.205	1.2139	1.2110	C2–O33	1.2105	1.2178

–C=O bond	C5–O13	1.2594	1.268	1.2596	1.2528	C4–O12	1.3116	1.3138

–C–C– bonds pyrrole ring	C3–C4	1.4536	1.451	1.4536	1.4529	C2–C3	1.4554	1.4530
	C4–C5	1.4113	1.394	1.4145	1.4116	C3–C4	1.3788	1.3836
	C5–C6	1.4807	1.483	1.4834	1.4898	C4–C5	1.4525	1.4552

–C–C– bonds carbon chain	C4–C7	1.4201	1.410	1.4260	1.4306	C3–C6	1.4489	1.4416
	C7–C9	1.5046	1.475	1.5057	1.5001	C6–C7	1.5001	1.4910
	C6–C14	1.3473	1.332	1.3470	1.3371	C5–C14	1.3516	1.3535
	C14–C16	1.4554	1.453	1.4557	1.4661	C14–C16	1.4521	1.4559

	C27–C29	1.5076	1.484	1.5078	1.4969	C27–C29	1.5064	1.4964

–C–C– bonds phenyl ring	C16–C25	1.4029	1.416	1.4029	1.3981	C16–C25	1.4036	1.4033
	C16–C17	1.4042	1.397	1.4042	1.3983	C16–C17	1.4049	1.4041
	C17–C19	1.3873	1.384	1.3874	1.3900	C17–C19	1.3868	1.3837
	C19–C21	1.3918	1.378	1.3918	1.3859	C19–C21	1.3919	1.3816
	C21–C23	1.3925	1.357	1.3925	1.3808	C21–C23	1.3928	1.3887
	C23–C25	1.3868	1.391	1.3868	1.3883	C23–C25	1.3860	1.3840

Ethanol	O34–C36	1.4362		1.4450	1.4543			
	C36–C39	1.5191		1.5167	1.4814			
	O34–H35	0.9647		0.96546	0.8403			

–M–O– bonds MO_6_ unit	M1–O13	1.9791	1.930	2.0574	2.0544			
	M1–O54	1.9791	1.930	2.0574	2.0544			
	M1–O8	1.9938	1.936	2.0782	2.0744			
	M1–O49	1.9938	1.936	2.0782	2.0744			
	M1–O34	2.4688	2.522	2.2323	2.1352			
	M1–O75	2.4688	2.522	2.2323	2.1352			

**Table 4:** Bond and copper valence data for Cu-complex.

*i*–*j*	*R*_*ij*_ (calc) [Å]	*S*_*ij*_ (calc)	*R*_*ij*_ (exp) [Å]	*S*_*ij*_ (exp)
Cu1–O13	1.9791	0.435	1.930	0.498
Cu1–O54	1.9791	0.435	1.930	0.498
Cu1–O8	1.9938	0.417	1.936	0.490
Cu1–O49	1.9938	0.417	1.936	0.490
Cu1–O34	2.4688	0.112	2.522	0.0962
Cu1–O75	2.4688	0.112	2.522	0.0962

		*S*_cu_ (calc) = 1.9		*S*_cu_ (exp) = 2.2

From these calculations a satisfactory agreement between *S*_cu_ (calc) and *S*_cu_ (exp) is stated.
